# Socioeconomic gradients in admission to coronary or intensive care units among Australians presenting with non-traumatic chest pain in emergency departments

**DOI:** 10.1186/s12873-018-0185-2

**Published:** 2018-09-29

**Authors:** George Mnatzaganian, Janet E Hiller, Jason Fletcher, Mark Putland, Cameron Knott, George Braitberg, Steve Begg, Melanie Bish

**Affiliations:** 1 La Trobe Rural Health School, College of Science, Health and Engineering, La Trobe University, PO Box 199, Bendigo, VIC 3552 Australia; 2 School of Health Sciences, Faculty of Health, Arts and Design, Swinburne University of Technology, John Street, Hawthorn, VIC Australia; 3 School of Public Health, The University of Adelaide, North Terrace, Adelaide, SA Australia; 4 Intensive Care Unit, Bendigo Health, Barnard Street, Bendigo, VIC Australia; 5 Department of Emergency Medicine, Royal Melbourne Hospital, Parkville, VIC Australia; 6 Monash Rural Health Bendigo, Monash University, Bendigo, VIC Australia; 7 Department of Intensive Care, Austin Health, Heidelberg, VIC Australia; 8 Centre for Integrated Critical Care Medicine, Department of Medicine and Radiology, The University of Melbourne, Parkville, VIC Australia

**Keywords:** Chest pain, Cardiovascular morbidity, Emergency department, Intensive care, Socioeconomic gradients

## Abstract

**Background:**

Socioeconomic inequalities in cardiovascular morbidity have been previously reported showing direct associations between socioeconomic disadvantage and worse health outcomes. However, disagreement remains regarding the strength of the direct associations. The main objective of this panel design was to inspect socioeconomic gradients in admission to a coronary care unit (CCU) or an intensive care unit (ICU) among adult patients presenting with non-traumatic chest pain in three acute-care public hospitals in Victoria, Australia, during 2009–2013.

**Methods:**

Consecutive adults aged 18 or over presenting with chest pain in three emergency departments (ED) in Victoria, Australia during the five-year study period were eligible to participate. A relative index of inequality of socioeconomic status (SES) was estimated based on residential postcode socioeconomic index for areas (SEIFA) disadvantage scores. Admission to specialised care units over repeated presentations was modelled using a multivariable Generalized Estimating Equations approach that accounted for various socio-demographic and clinical variables.

**Results:**

Non-traumatic chest pain accounted for 10% of all presentations in the emergency departments (ED). A total of 53,177 individuals presented during the study period, with 22.5% presenting more than once. Of all patients, 17,579 (33.1%) were hospitalised over time, of whom 8584 (48.8%) were treated in a specialised care unit. Female sex was independently associated with fewer admissions to CCU / ICU, whereas, a dose-response effect of socioeconomic disadvantage and admission to CCU / ICU was found, with risk of admission increasing incrementally as SES declined. Patients coming from the lowest SES locations were 27% more likely to be admitted to these units compared with those coming from the least disadvantaged locations, *p* <  0.001. Men were significantly more likely to be admitted to such units than similarly affected and aged women among those diagnosed with angina pectoris, arrhythmia, myocardial infarction, heart failure, chest pain, and general signs and symptoms.

**Conclusions:**

This study is the first to report socioeconomic gradients in admission to CCU / ICU in patients presenting with chest pain showing a dose-response effect. Our findings suggest increased cardiovascular morbidity as socioeconomic disadvantage increases.

## Background

Socioeconomic inequalities in cardiovascular morbidity and mortality have been reported in many regions including the US [1], the UK [2], Australia [3] and other Organisation for Economic Cooperation and Development (OECD) countries [4]. Cardiovascular morbidity measures such as admission rates for cardiac related conditions have generally dropped over the past two decades [5]. However, relative inequality in cardiovascular emergency admissions and cardiovascular related mortality actually increased in the most disadvantaged compared with the least disadvantaged [6]. These disparities have been attributed to a range of socioeconomic determinants of health and health behaviours, rooted in social rank as determined by education, occupational hierarchy and income [1, 4, 7, 8]. The relationship between socioeconomic status and general health, and, in particular, cardiovascular health, has been demonstrated within different races and ethnic groups [9], suggesting that cultural and ethnic dissimilarities do not explain the socioeconomic differences. These associations were also observed in countries with universal access to health care [2, 3], and when comparing those who have similar rates of smoking, obesity and alcohol use [9].

Despite the mounting evidence of direct associations between socioeconomic disadvantage and poorer health outcomes, disagreement remains regarding the strength of the evidence to support causality [10–14] with reverse causation proposed as a contributor to the relationship. For example, illness may lead to lower academic achievement or loss in income [10]. Studies investigating these relationships have predominantly followed a cross-sectional design and have not been population-based limiting their generalisability [13]. Furthermore, no study found dose-response associations between lower SES and increased cardiovascular morbidity.

Chest pain is a frequently seen symptom in emergency departments, being the most common reason for presenting in the ED among Americans over the age of 65 and contributing to approximately 6 million visits per annum under the US Medicare system [15]. Clinical management of chest pain is highly variable, often depending on the underlying causes and is considered a medical emergency until all life-threatening causes have been ruled out. Potentially life-threatening causes of chest pain include acute myocardial infarction and other acute coronary syndromes as well as aortic dissection, pneumothorax, pneumonia and pulmonary embolism. Patients presenting with chest pain who require critical care are often more likely to be severely ill and / or are at risk of imminent death [16, 17]. This study used emergency admissions following repeated non-traumatic chest pain presentations to emergency departments (ED) to explore the associations between sex, age, geographic-based socioeconomic disadvantage score and cardiovascular morbidity, expressed as admission to specialised care units.

## Methods

### Study population

Consecutive adults aged 18 or over presenting with chest pain (including chest heaviness, heart pain, and chest tightness) in three emergency departments in Victoria, Australia during January 2009 and December 2013 were eligible to participate in this population-based panel study. Cases were identified using the International Classification of Diseases, 10th Revision, (Australian Modification) (ICD-10-AM) code of R07. Patients presenting with chest pain due to trauma or other injury were excluded from this analysis.

All three hospitals serve different catchment populations in metropolitan Melbourne, Australia, with a combined total of more than 200,000 ED presentations per annum [18]. The hospitals differ by their bed capacity, with Hospital A being the smallest and Hospital C the largest.

### Study variables

Sociodemographic and presentation-related variables together with clinical variables were collected from the ED electronic database (SYMPHONY Version 2.29). The collected information included age, sex, country of origin, residential postcode, main language spoken at home, arrival mode, arrival time, presenting symptoms, a registered nurse-allocated triage urgency score that categorised the presentation as being an emergency (triage scores of 1 or 2), urgent (triage score of 3) or semi and non-urgent (triage scores of 4 or 5) and length of stay in the ED. The final main acute diagnosis reached on discharge from the ED was also collected together with the discharge destination. Among admitted patients, three possible admission departments were recorded: CCU, ICU and medical ward. The medical ward was used to categorise all adult hospital departments that did not have intensive specialised care. The admitting ward of patients transferred to another public or private hospital was also recorded and accounted for in this analysis. All diagnoses were identified using ICD-10-AM codes.

Each individual’s residential postcode was merged with the Australian Bureau of Statistics to obtain the Socio Economic Index For Areas (SEIFA) [19], estimated from the 2011 census data. SEIFA is a composite index of relative advantage or disadvantage based on geographic areas across Australia, with higher scores indicating less socioeconomic disadvantage. The SEIFA was further used to calculate a Relative Index of Inequality (RII) which is a regression-derived index summarising the magnitude of socioeconomic disadvantage while taking into account the sample size and the relative disadvantage experienced by each individual [20]. The estimated RII was further introduced as quintiles categorised according to the score’s distribution in the sample.

### Statistical analysis

Patient characteristics were compared by study categorical variables using Pearson Chi-square while ANOVA tests compared the means. The multivariable analyses were conducted on the first presentation [Model 1] (irrespective of study outcome) and on all repeated presentations [Model 2] during the study five-year period. A logistic regression was used to model admission to these units on the first presentation. The dose-response effect of different levels of relative index of socioeconomic inequality on admission to specialised care was tested using log likelihood ratio tests which evaluated linear trends. An insignificant *p* value of the log likelihood test indicated linearity.

Admission to CCU or ICU over the five-year study period was analysed using the Generalized Estimating Equations (GEE) approach [21]. An exchangeable working covariance matrix was used to account for correlation and dependence between repeated measurements on the same individual over time while accounting for: age, sex, relative index of socioeconomic inequality, country of origin, main language spoken at home, ambulance arrival mode, arrival time, presenting symptoms, nurse allocated triage urgency score, treating hospital, length of stay in the ED, and main diagnosis on discharge from the ED.

In a sub analysis, the multivariable regression was further run on only patients who were admitted to hospital.

Stata statistical program (version 15, StataCorp, College Station, TX, USA) was used to conduct the analyses.

## Results

### Descriptive

**Fig. 1:**
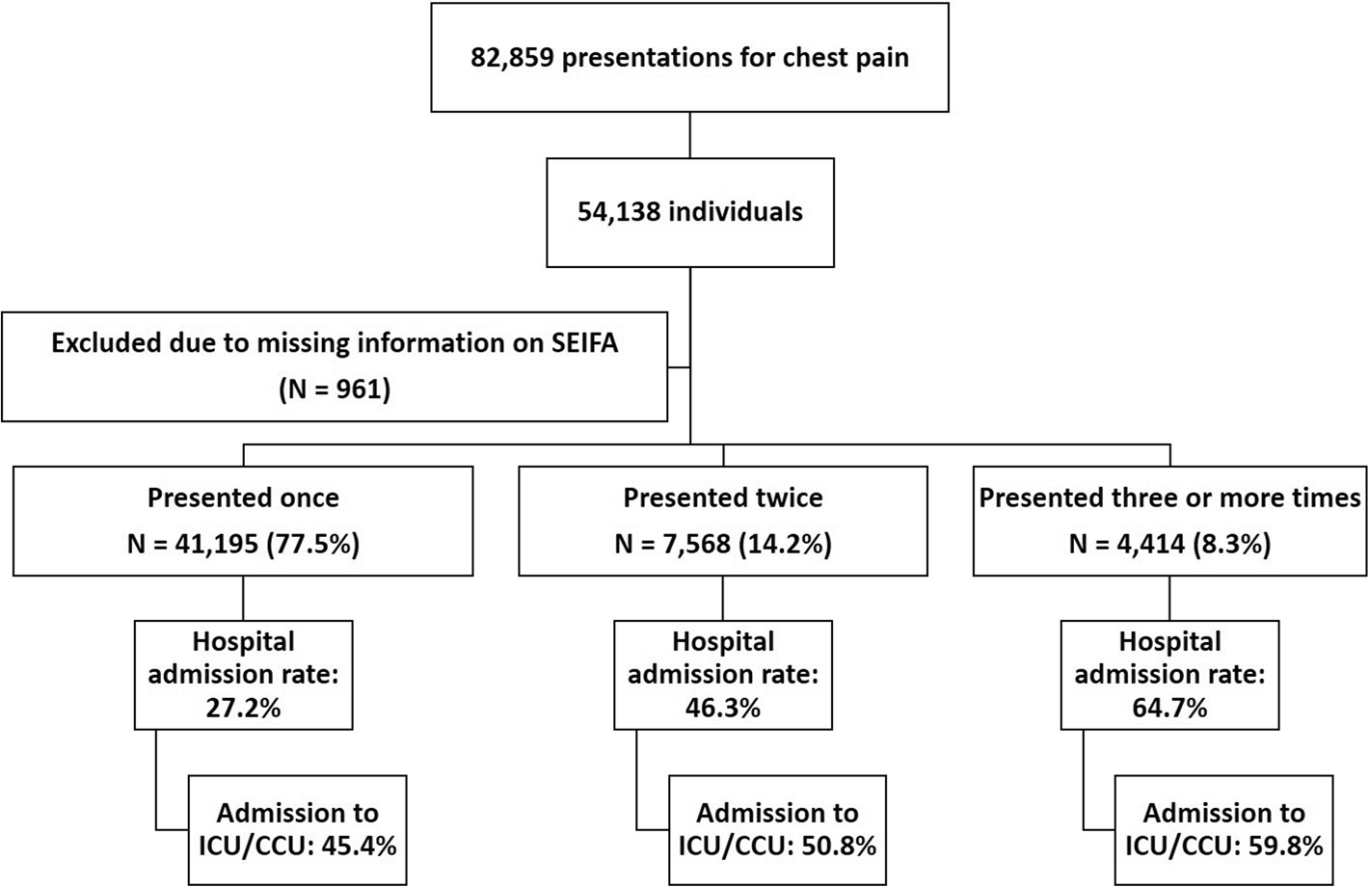
Flow chart of study participants

During the study’s five-year period, a total of 82,859 chest pain presentations were recorded, for a total of 54,138 individuals, being approximately 10% of all presentations in the EDs of the three hospitals combined. Of these, 961 (1.8%) were excluded because of missing information on residential postcode (Fig. 1). Of the remaining 53,177 individuals, 77.5% presented once, 14.2% presented twice, and 8.3% presented three or more times. Hospital admission rates significantly rose with increased number of presentations (*p* <  0.001); admission to a CCU or ICU similarly rose with increased presentations (*p* <  0.001) (Fig. 1). The geographic-based socioeconomic groups considerably differed on age, country of birth, spoken language at home, and presentation characteristics (Table 1). Patients coming from low SES locations tended to repeatedly present with chest pain during the five-year study period (*p* <  0.001) (Table 2). Such patients also tended to receive lower urgency triage scores on arrival to the ED. However, except for those coming from the least disadvantaged locations, crude admission rates to CCU or ICU were similar in all other sub-groups (Table 1), with no evidence for trend observed.

**Table 1:** Patient characteristics and emergency department visit outcome on first presentation by quintiles of the relative index of inequality of socioeconomic disadvantage

	All *N* = 53,177	RII_1 Highest SES *N* = 10,815	RII_2 N = 10,666	RII_3 N = 10,994	RII_4 N = 10,831	RII_5 Lowest SES *N* = 9871	*P* value^
Median age of study sample,%							
53 years or younger	50.4	49.3	52.3	49.7	54.0	46.5	< 0.001
54 years or older	49.6	50.7	47.7	50.3	46.0	53.5	
Female sex, %	48.8	49.3	49.2	48.5	49.1	47.9	0.2
Region of birth,%							
Oceania	55.1	58.7	60.0	59.7	56.7	39.3	< 0.001
Europe / Americas	23.8	22.0	21.4	24.9	21.3	29.7	
Asia / Middle East	15.8	14.2	13.6	10.9	16.7	24.5	
All other	5.3	5.1	5.1	4.6	5.3	6.4	
English spoken at home, %	90.4	94.0	94.0	92.0	91.5	79.8	< 0.001
Arrival to ED by ambulance, %	44.0	40.3	46.8	45.8	43.7	43.4	< 0.001
Nurse Triage presentation urgency score, %							
Resuscitation / Emergency	44.6	46.2	45.2	44.8	43.9	42.9	< 0.001
Urgent	43.5	42.6	43.4	43.5	43.7	44.3	
Semi-urgent / Non-urgent	11.9	11.2	11.4	11.7	12.4	12.8	
ED visit outcome, %							
Admitted to CCU / ICU	13.2	11.8	13.4	13.8	13.7	13.4	< 0.001
Admitted to a medical ward	15.9	16.1	16.6	15.8	14.9	16.1	
Died in the ED	0.4	0.4	0.6	0.3	0.4	0.5	
Discharged home	70.5	71.8	69.4	70.1	71.0	69.9	

*Abbreviations*: *CCU* coronary care unit, *ED* emergency department, *ICU* intensive care unit, *RII* relative index of inequality, *SES* socio-economic status

^ Proportions were compared using chi-square tests and means were compared using one-way ANOVA tests

**Table 2:** Number of chest pain presentations over study five-year period by quintiles of the relative index of inequality of socioeconomic disadvantage

	All *N* = 75,105	RII_1 Highest SES *N* = 14,551	RII_2 N = 14,578	RII_3 *N* = 15,170	RII_4 *N* = 15,318	RII_5 Lowest SES N = 15,488	*P* value
Presented once	54.9	59.1	58.4	56.7	53.9	46.6	< 0.001
Presented twice	19.4	19.5	18.2	19.1	20.6	19.4	
Presented three or more times	25.8	21.4	23.4	24.2	25.5	34.0	

Of the 53,177 presenting individuals, 17,579 (33.1%) were hospitalised over time, of whom 8584 (48.8%) received specialised care. Compared to men, presenting women were more likely to be discharged home, and those admitted were less likely to be treated in a specialised care unit. Sex differences were mainly observed among those diagnosed with myocardial infarction, heart failure, angina pectoris, arrhythmia, chest pain, and general signs and symptoms. In all these diagnoses, men were significantly more likely than women to be admitted to a specialised care unit, *p* <  0.001 in each. Proportion of males admitted to specialised care units was higher than those of females shown in all age categories (Table 4 in Appendix).

### Multivariable analyses

In the logistic multivariable regression [Model 1], younger age and female sex were independently associated with fewer admissions to a CCU or ICU, whereas, patients coming from low SES locations were more likely to be admitted to such units compared to those coming from the least disadvantaged locations. Likelihood ratio tests to investigate linear associations showed a dose-response effect of geographic-based socioeconomic disadvantage and admission to a specialised care unit, with risk of admission increasing incrementally as socioeconomic disadvantage increased (Likelihood-ratio Chi-square = 0.6, *p* = 0.897) (Table 3).

**Table 3:** Multivariable regressions^b^ investigating risk of admission to a coronary care unit or intensive care unit among patients presenting with non-traumatic chest pain in emergency departments: 2009–2013

Model^a^	Covariates	Adjusted-OR	95% CI	*P* value
1	Age (continuous)	1.00	1.00–1.01	< 0.001
	Female sex	0.58	0.54–0.61	< 0.001
	Relative index of SES inequality quintiles			
	1st quintile (Highest SES)	1.00		
	2nd quintile	1.10	0.99–1.22	0.054
	3rd quintile	1.14	1.02–1.26	0.017
	4th quintile	1.24	1.12–1.36	< 0.001
	5th quintile (Lowest SES)	1.33	1.21–1.47	< 0.001
	*Testing linear trends for RII: Likelihood ratio test Chi-square = 0.6, p = 0.897*			
2	Age (continuous)	1.00	1.00–1.01	< 0.001
	Female sex	0.61	0.57–0.64	< 0.001
	Relative index of SES inequality quintiles			
	1st quintile (Highest SES)	1.00		
	2nd quintile	1.07	0.99–1.17	0.1
	3rd quintile	1.07	0.97–1.16	0.2
	4th quintile	1.18	1.08–1.28	< 0.001
	5th quintile (Lowest SES)	1.27	1.17–1.39	< 0.001

^a^ Model 1: Logistic regression that modelled risk of admission to CCU or ICU on first presentation

Model 2: Generalized Estimating Equations regression that modelled risk of admission to CCU or ICU including all repeated presentations during study five-year period

^b^ Both multivariable models were also accounted for: region of birth, language spoken at home, mode of arrival, time of arrival, hospital type, nurse triage urgency score, symptoms on arrival, length of stay in the ED, and main acute admission diagnosis

The GEE multivariable model [Model 2], that accounted for repeated presentations over time, showed identical results. Females were 39% less likely than males to be admitted to a specialised care bed (Adjusted-OR = 0.61, 95% CI 0.57–0.64, *p* <  0.001). Patients coming from the lowest SES locations were 27% more likely to be admitted to these units compared with the least disadvantaged category, (Adjusted-OR = 1.27, 95% CI 1.17–1.39, *p* <  0.001) (Table 3). Other covariates associated with increased admission to ICU or CCU included arrival by ambulance, higher presentation urgency scores, altered consciousness on presentation, arrival time (with highest rates seen during the evening hours), and acute diagnoses such as acute myocardial infarction.

A sub-analysis that was limited to patients who were admitted to the hospital showed similar results except for age. Among admitted patients, older patients were less likely than their younger counterparts to be admitted to ICU or CCU.

## Discussion

In this large population-based panel analysis that investigated emergency non-traumatic chest pain admissions, geographic-based socioeconomic disadvantage scores were independently associated with increased admissions to a coronary or intensive care unit, showing a dose-response. These associations were independent of the patient’s sex, age, ethnicity, main presenting symptoms, hospital setting, urgency of presentation, length of stay in the ED, and main acute admission diagnosis. Females compared to males of the same age were less likely to be admitted to such units.

The findings of this study suggest increased chest pain severity or increased general and, in particular, cardiovascular morbidity among those coming from low SES locations. However, although this study reports dose-response effect between socioeconomic disadvantage and admission to intensive or coronary care units, and despite the mounting and consistent findings of direct associations [4, 8, 9, 11], this study was observational, and a causal relationship between socioeconomic disadvantage and worse health outcomes cannot be inferred. The relationship between socioeconomic disadvantage and health is complex and often indirect involving multiple, temporally-evolving relationships between biological, behavioural, psychological, sociological or environmental factors [22]. Residual confounding by many factors not accounted for in this study cannot be excluded [23]. The found inverse associations could possibly be explained by unmeasured factors un-accounted for in this current study. Some of such factors relate to obesity and to smoking. Individuals coming from more disadvantaged backgrounds often smoke more and are more likely to have tobacco-related comorbidities that impact cardiovascular morbidity [24]. Similarly, the relationship of obesity with heart disease, hypertension, some cancers, type 2 diabetes and stroke are well established [25]. However, the association of obesity with socioeconomic disadvantage is multifaceted. Although obesity has been rising across all social classes, research indicates that some groups are more vulnerable than others. This variation of obesity by socioeconomic classes is complex as highlighted by a study that measured prevalence of obesity by different socioeconomic groups in 67 countries [26]. These authors show that in low-income and developing economies the affluent are more likely to be obese; however, a reverse relationship is noted in rich and developed economies where obesity is more prevalent among those coming from lower socioeconomic classes.

This current study also reports significant associations relating to age and sex – findings that may have ramifications for critical care services. As the population ages, the proportion of patients in need of critical care resources will likely increase. A six-year retrospective study that investigated 57 ICUs in Australia and New Zealand reported a significant rise in admission rates among patients aged 80 years or older of nearly 6% per year [27]. A similar rise in demand for critical care by the elderly has been reported in other countries as well [28]. In the United States, over the past ten years, the number of ICU beds per adult population mainly grew in regions with the largest elderly populations [28], with approximately 55% of all intensive care beds being occupied by patients aged 65 or more with 14% of patients 85 or more dying in the ICU setting. A Dutch study, that compared 1996 with 2006, found a 33% increase in number of patients aged 75 or more that needed critical care [29]. This rise in demand for critical care has been reported in North America, Europe, Australia and other regions, but not all patients meeting critical care admission criteria are admitted to such facilities [30, 31]. A British study that investigated ICU admissions among surgical patients in 94 National Health Service hospitals between 1999 and 2004 reported that approximately 85% of the surgical patients who died during admission were never admitted to an intensive care facility [30]. An Israeli incidence study found that 55% of all critically ill hospitalised patients were treated in medical wards that had no intensive specialised care [31]. Similar to these authors who reported that older age was associated with less admissions to an ICU, our study found that among the hospitalised population, older patients were less likely than younger patients to be admitted to a critical care unit. Given higher prevalence of comorbidities and frailty in the elderly, a less favourable risk profile for adverse outcomes and mortality may render them unfit to be admitted to a specialised care unit. This prognosis-based selection bias is commonplace when the demand for these specialised beds far exceeds their availability [31].

Sex variation in hospital admission rates among those presenting to the ED with coronary syndromes has also been previously reported by many studies [32–35]. In general, women take longer than men to seek medical advice for chest pain [33], and when they do, compared to men diagnosed with similar conditions, women are less likely to be admitted to an acute care hospital and to undergo coronary revascularisation. Admitted women are also less likely to be treated in a specialised care unit. A two-year retrospective Canadian study found sex- and age-specific differences in ICU admission rates observed in over 24,000 consecutive adult ICU admissions [36]. A large three-year prospective European study showed that women admitted to critical care units were less likely than their male counterparts to receive invasive therapy and to have shorter ICU durations of stay, despite women having higher severity of illness scores than men needing admission to ICU [34]. The authors also reported a significantly higher risk adjusted in-hospital and ICU mortality among women. Age may be one explanation, as women are often older than men when diagnosed with a serious cardiac condition such as AMI. Compared to men, women also undergo fewer invasive procedures which might have lowered their chances of being admitted to an ICU or CCU as such admissions are higher following major procedures and surgeries [37, 38]. Our study and others [34, 36] show that age and acute diagnoses such as AMI cannot explain the sex differences in hospital care following a chest pain presentation in the ED.

### Strengths and limitations

This study utilised a large population-based dataset that included all emergency non-traumatic chest pain presentations in three Victorian hospitals that serve uniquely different sub-populations. The study population was limited to those presenting with emergency non-traumatic chest pain. This has subsequently minimised elective admissions, such as for coronary angioplasty where the highest rates are reported in the more affluent. Compared to the latter, patients coming from socioeconomically disadvantaged backgrounds often wait longer for, and have less access to, a coronary angioplasty consistently observed in the US, UK, and Australia [5, 38]. The admitting ward of patients transferred to other hospitals was also captured in this analysis; for example, if patients coming from higher SES locations chose to be transferred to other private hospitals.

The study is limited primarily by the available data and the ability to account for comorbidities, obesity, severity of illness, actual geographic distances that the patients needed to travel to reach the ED, and health behaviours including smoking and physical exercise. Although comorbidities may affect medical outcomes [39] and influence clinical decision-making for CCU or ICU admission, the analysis found sex-specific differences in all age groups and not specifically in the older patients who are more likely to have multiple comorbidities. Higher admission rates among patients coming from lower SES locations may indicate more morbidity and more severity of illness, as specialised care units are more likely to admit those with greater illness severity [31, 40]. CCU / ICU bed availability was not known; however, availability of beds cannot explain the risk-adjusted differences found. We had no information on patients’ level of education or income and we used an ecological index to infer social status. The use of the composite variable of SEIFA could have misclassified the true socioeconomic status. The study was confined to the three participating hospitals. Finally, the effect of transfer between clinical care wards was not known as no information on inter-departmental movements was known.

## Conclusions

To our knowledge, this study is the first to report dose-response relationships between geographic-based socioeconomic disadvantage and higher admission rates to specialised care units in a population-based representative sample of patients presenting with emergency chest pain. The relationship between socioeconomic disadvantage and cardiovascular morbidity is complex and multifactorial. Not every individual exposed to lower socioeconomic disadvantage develops disease and our results cannot infer causal relationships but our findings add to the accumulating evidence supporting a direct association between socioeconomic disadvantage and increased morbidity.

## Appendix

**Table 4:** Percent admitted to CCU or ICU by age and sex

Age categories in years	% Male admitted to CCU / ICU	% Female admitted to CCU / ICU	*P* value
24 or younger	29.0	19.1	0.09
25–29	39.8	21.8	0.008
30–34	46.7	32.5	0.018
35–39	56.3	31.6	< 0.001
40–44	51.6	37.6	< 0.001
45–49	60.8	36.9	< 0.001
50–54	59.9	46.7	< 0.001
55–59	58.6	49.4	0.001
60–64	56.9	48.5	0.001
65–69	58.0	42.3	< 0.001
70–74	51.6	41.8	< 0.001
75–79	47.6	40.5	0.003
80–84	38.9	31.2	0.001
85 or older	28.6	16.6	< 0.001
All ages together	51.6	36.7	< 0.001

*Abbreviations*: *CCU* coronary care unit, *ED* emergency department, *ICU* intensive care unit

## Abbreviations

AMI: Acute myocardial infarction
CCU: Coronary care unit
ED: Emergency department
GEE: Generalized Estimating Eqs.
ICD-10-AM: The International Classification of Diseases, 10th Revision, (Australian Modification)
ICU: Intensive care unit
RII: Relative Index of Inequality
SEIFA: Socio Economic Index For Areas
SES: Socioeconomic status
